# Arabic Captioning for Images of Clothing Using Deep Learning

**DOI:** 10.3390/s23083783

**Published:** 2023-04-07

**Authors:** Rasha Saleh Al-Malki, Arwa Yousuf Al-Aama

**Affiliations:** Computer Science Department, Faculty of Computing and Information Technology, King Abdulaziz University, Jeddah 21589, Saudi Arabia; aalaama@kau.edu.sa

**Keywords:** deep learning, image captioning, transfer learning, image attributes

## Abstract

Fashion is one of the many fields of application that image captioning is being used in. For e-commerce websites holding tens of thousands of images of clothing, automated item descriptions are quite desirable. This paper addresses captioning images of clothing in the Arabic language using deep learning. Image captioning systems are based on Computer Vision and Natural Language Processing techniques because visual and textual understanding is needed for these systems. Many approaches have been proposed to build such systems. The most widely used methods are deep learning methods which use the image model to analyze the visual content of the image, and the language model to generate the caption. Generating the caption in the English language using deep learning algorithms received great attention from many researchers in their research, but there is still a gap in generating the caption in the Arabic language because public datasets are often not available in the Arabic language. In this work, we created an Arabic dataset for captioning images of clothing which we named “ArabicFashionData” because this model is the first model for captioning images of clothing in the Arabic language. Moreover, we classified the attributes of the images of clothing and used them as inputs to the decoder of our image captioning model to enhance Arabic caption quality. In addition, we used the attention mechanism. Our approach achieved a BLEU-1 score of 88.52. The experiment findings are encouraging and suggest that, with a bigger dataset, the attributes-based image captioning model can achieve excellent results for Arabic image captioning.

## 1. Introduction

The computer vision research community has concentrated on fashion-related research over the past few years due to the expansion of e-commerce websites and the rising significance of fashion and style in our everyday lives. And as accurate and attractive descriptions of clothing on shopping websites might help customers better comprehend the attributes of clothing and increase online sales by drawing in more users, researchers show a lot of interest in multi-modal tasks such as image captioning [1]. Image captioning is a fundamental job in artificial intelligence that uses natural language to describe the attributes, objects, and relationships in an image. It may be used for a variety of purposes, including semantic image search, image indexing, giving chatbots visual intelligence, and assisting those who are visually impaired to see the world around them and to comprehend content of images [2].

Understanding visual and textual content is needed for image captioning systems, where Computer Vision and Natural Language Processing techniques are what these systems are based on. Research related to generating the caption of an image in the English language has grown significantly. However, research on generating captions in the Arabic language is limited, because there are not as many robust datasets for the Arabic language as there are for English.

Several automatic Arabic image captioning systems have been developed using deep learning and several approaches have been used in these systems. The approach of using Convolutional Neural Networks (CNN) to process the images and Recurrent Neural Networks (RNN) to process the caption was proven to be the most successful and achieved fairly good results [3]. In addition, the attention mechanism has been used by researchers in English image captioning and has shown to give good results [4]. However, it has not been used in Arabic captioning attempts. In this work, we provide a public dataset for captioning images of clothing in the Arabic language. Also, we decided to use the attention mechanism to improve performance.

The remainder of this paper is organized as follows. Section 2 summarizes the literature review related to this work. Our proposed method is detailed in Section 3. The experimental setup is reported in Section 4. Results and discussion are presented in Section 5. Finally, Section 6 concludes the paper.

## 2. Literature Review

In this section, first, we discussed image captioning approaches. Second, we presented an overview of the transfer learning technique. Third, we discussed Arabic image captioning approaches for generating the caption for an image in the Arabic language. Fourth, we discussed different datasets used for captioning images of clothing. Fifth, images of clothing captioning approaches in the English language.

### 2.1. Image Captioning Approaches

Image captioning can be done in different ways. We listed the most significant techniques based on the model architecture, image model, language model, and attention mechanism.

#### 2.1.1. Model Architecture

An Encoder-Decoder architecture for image captioning was used by Vinyals et al. [5]. They used a Convolutional Neural Network (CNN) as an encoder to extract image features. Then the extracted feature vector is sent to a decoder to generate a caption word by word.

**Figure 1:**
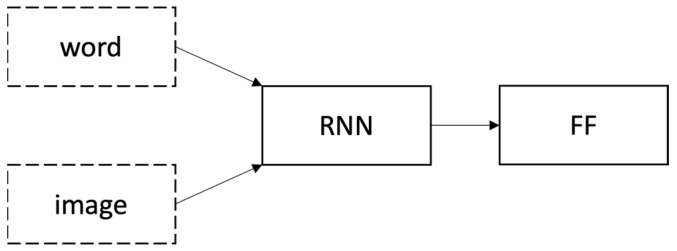
Inject architecture [6].

**Figure 2:**
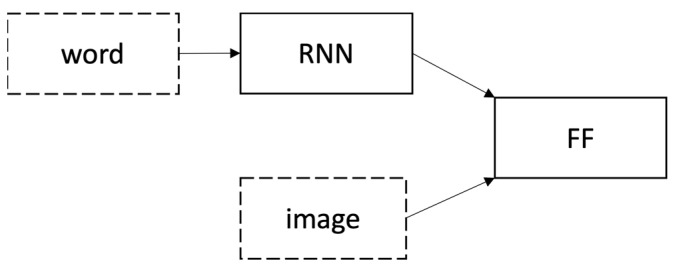
Merge architecture [6].

Based on Tanti et al. [6], inject architecture and merge architecture are two widely used architectures for encoder-decoder models among deep learning approaches when captions are generated using a Recurrent Neural Network (RNN) as a language model. Inject architectures are based on injecting image features into the same RNN that processes the words, i.e., RNN is considered as ‘generators’. A depiction of an inject architecture is shown in Figure 1. However, a merge architecture is based on merging image features with the RNN’s final state after processing the words (linguistic features) in a separate multimodal feed-forward layer (FF) that drives the generation process, i.e., RNN is considered as a ‘linguistic encoder’. Merge architecture is shown in Figure 2.

After comparing these two architectures, the findings showed that the merged architecture outperformed the injection architecture in performance [6].

#### 2.1.2. Image Model

According to [7], the first part of a caption-generating model is an image model. It works as an encoder, transforming an input image, which is a vector of three elements (RGB), into a feature vector of learned elements. One of the Deep Neural Network (DNN) classes that are frequently employed for image captioning and visual content analysis is the Convolutional Neural Network (CNN). Several CNN models may be used to extract image features; each one has a different number of layers and filters but shares the same basic architecture as follows:

- Convolutional layer: The most important component. It is comprised of a number of convolutional filters called kernels. These filters are used to extract the features of the input image to generate the output feature map [8].
- Pooling layer: Reduces the size of large feature maps that were produced through the use of convolutional operations, to create smaller feature maps. At the same time, it keeps most of the dominating information (or features) during the whole pooling stage. Maximum, minimum, and average pooling are the most popular and commonly used pooling methods.
- Fully connected layer: This layer is the last layer of each CNN architecture. According to the so-called Fully Connected (FC) approach, every neuron in this layer is connected to every neuron in the previous layer. It takes the input from the last pooling or convolutional layer in the form of a vector and applies an activation function [8].

Alam et al. [9] compared five popular CNNs architectures used as image captioning encoders. They used Long Short-Term Memory (LSTM) as decoder architecture using the inject architecture for image captioning. The networks compared were ResNet50, VGG16, Densenet201, InceptionV3, and Xception model. They used categorical cross-entropy to measure loss function and Root Mean Squared Propagation (RMSprop) as an optimizer to optimize the models. RMSProp is an optimization algorithm designed for neural networks. They showed the training’s accuracy and value loss. According to their research, Resnet50 provided better accuracy.

- ResNet50

**Figure 3:**
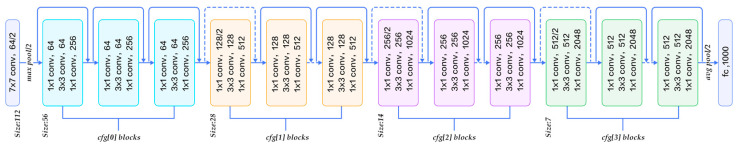
ResNet50 network architecture [10].

Residual Neural Network (ResNet) was developed by Kaiming He et al. [10] and had a 3.57% error rate, which was lower than the previous networks. It was utilized for classification and recognition tasks. ResNet50 pre-trained model has 50 layers, although it has also been developed with different numbers of layers, including 18, 34, and 152. ResNet50 consists of 49 convolution layers and a fully connected layer. It comprises five blocks of convolution layers, referred to as Block1 through Block5, and a fully connected layer containing 1000 nodes and a softmax function at the end, which uses global average pooling to improve classification accuracy while reducing the number of parameters. The basic architecture of the ResNet50 layers is illustrated in Figure 3.

#### 2.1.3. Language Model

To encode linguistic sequences, many approaches were used. The language models that are most frequently used for image captioning are:

- Recurrent Neural Networks (RNNs): RNNs are more suitable for tasks requiring sequence learning because their nodes can build a directed graph and make use of internal state (memory) to process sequences of inputs. The vanishing and exploding gradient issue prevent traditional RNNs from predicting words in long-range dependencies. Hence, an improved version of RNN called Long Short-Term Memory (LSTM) is designed [7].
- Long Short-Term Memory (LSTM): An improved version of RNN consists of a memory cell to maintain information in the memory for long periods of time. The information flow in the LSTM model is controlled by an internal mechanism known as gates. In order to pass the data required to predict the sequence chain, the gates determine which data to keep and which to forget. It consists of three windows: an input gateway, an output gateway, and a forget gateway [11].

#### 2.1.4. Attention Mechanism

The attention mechanism was proposed by Kelvin et al. [12] which dynamically focuses on the salient parts of the image and generates the associated words during the decoding process. The use of attention results in a higher score and better performance. In the field of deep learning, attention has become one of the most influential concepts. It is inspired by human biological systems, which, while processing large amounts of information, tend to focus on the distinctive parts. The attention mechanism has been widely used in a variety of application domains with the development of deep neural networks [4].

### 2.2. Transfer Learning

Transfer learning is a machine learning method in which a model that has been trained for a specific task is used for another task. Transfer learning solves such cross-domain learning difficulties by transferring relevant knowledge from data in a related domain to use in the target tasks. It has gained popularity and become a promising field in machine learning and deep learning as a result of its broad application potential [13].

### 2.3. Arabic Image Captioning Approaches

The best and most current approaches for generating the caption in the Arabic language will be discussed here.

Jindal [14] proposed one of the first deep learning approaches for generating Arabic captions. His approach used root words Recurrent Neural Network (RNN) and Deep Belief Network (DBN) to generate root words rather than actual sentences or descriptions. DBN is an algorithm for unsupervised probabilistic deep learning, which is a deep architecture-based multi-layer generative graphical model. DBNs have top-down connections in the bottom layers, whereas the top layer has bidirectional connections. Jindal’s methodology for image captioning includes three main steps: first, fragments of the image are extracted to extract the features by a Regional Convolutional Neural Network (R-CNN) which divides the image into 2000 regions and feeds the CNN with each region. Second, each image fragment is mapped to Arabic root words using pre-trained DBNs. Finally, the full caption with words in the correct order using the dependency tree relation is generated. He used Arabic translators to translate 10,000 images from the imageNet dataset, and he collected 100,000 images from AlJazeera News website with its Arabic caption. His technique achieved a BLEU-1 score of 34.8.

Mualla et al. [15] developed an Arabic image description system. To generate captions, they used the inject model, where image features extracted from a CNN and image description files are injected into the same RNN which generates the captions. They translated a subset of the Flickr8k dataset (2000 images) into the Arabic language and used them for training, validation, and testing their model (1500, 250, 250) respectively. Their model achieved a BLEU-1 score of 34.4.

An image captioning model for generating Arabic captions has also been proposed by Al-Muzaini et al. [3]. Their proposed model uses a merged model in which image features and captions are learned independently. The model is composed of three parts. First, an RNN with LSTM to build a language model to encode the varying length of linguistic sequences is used. Second, a CNN to build an image feature extractor model to extract features from images in the form of a vector with a fixed length is used. Third, a decoder model to make a final prediction, which takes the outputted vectors from the previous models as input is used. They used crowdsourcing to build an Arabic version of the MSCOCO dataset (1166 images), Arabic translators, and Google translation API to translate parts of the Flicker8k dataset to Arabic (2261 images). Their model achieved a BLEU-1 score of 46 which outperformed the models in [14,15].

Jindal [16] extended his work in [14]. Where in the second step, instead of generating root words for an image in Modern Standard Arabic (MSA) using Deep Belief Networks, he used a word-based RNN with LSTM. He used Arabic translators to translate the Flickr8k dataset to Arabic, and 405,000 images with captions from newspapers from various Middle Eastern countries. His approach achieved a BLEU-1 score of 65.8 on the Flickr8k dataset which is the best BLEU-1 score, and a BLEU-1 score of 55.6 on the Middle Eastern News Websites dataset.

ElJundi et al. [17] also proposed a sequence-to-sequence encoder-decoder framework by using the inject model for generating Arabic captions. They translated the Flickr8k dataset (8092 images) and used them for training, and testing their model (7292, 800) respectively. They were the first to make an Arabic dataset publicly available for researchers to use. Their model achieved a BLEU-1 score of 33.

Table 1 summarizes existing Arabic image captioning approaches.

As can be seen, all the developed Arabic image captioning models depended on generating the caption by considering the scene as a whole and not using the attention mechanism.

### 2.4. Dataset Collection

We present the datasets we found that are used for captioning images of clothing in the English language.

- DeepFashion dataset: A large-scale clothing dataset built with four benchmarks for evaluating different methods. It is developed by Liu et al. and it consists of 800,000 images with attributes and captions. Each image in this dataset is labeled with 50 categories, 1000 descriptive attributes, and clothing landmarks. The images of clothing were collected from image search engines, where the resulting images come from blogs, forums, and other user-generated content, which supplement and extend the image set collected from the shopping websites [18].
- Fashion MNIST dataset: It contains 70,000 fashion items across 10 different categories, with 7000 pictures in each category. It is divided into 60,000 for training, while the remaining are for testing. This dataset is a dataset of Zalando’s article images [1].

### 2.5. Images of Clothing Captioning

Since the use of deep learning-based Arabic image captioning in the field of clothing was not found during our review of the literature, the most prominent approaches for captioning images of clothing in the English language will be discussed.

**Figure 4:**
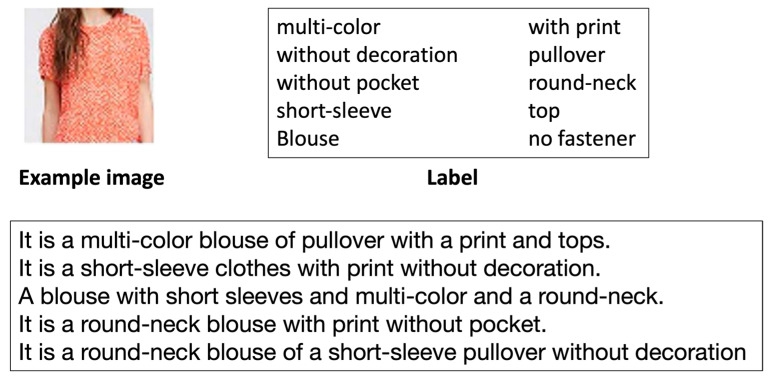
Image of clothing, labels, and descriptive caption examples [19].

A clothing caption-generating-based system was developed by Tateno et al. [19]. It depends on Deep Neural Networks (DNNs) to transform visual information obtained from clothing into verbal representation. They created a dataset for images of clothing, and it was limited to tops, where they selected images from the DeepFashion dataset and regenerated the caption for it. The dataset contains 20,096 images with five captions for each image; and in the caption, they used sentences with only objective representation. They labeled all images of clothing based on 10 items (fasteners, pockets, collars, color, print, decoration, wear, attributes, tops, and sleeves) and they created captions from these labels. Figure 4 shows an example. The image features were extracted using a pre-learned VGG16, which are then fed with the caption into LSTM without the use of a word embedding layer. They tested their model only on 100 unseen images. Their model generated correct captions for 80% of these images.

Hacheme et al. [20] addressed dataset diversity issues in image captioning for images of clothing. They built an African fashion dataset (InFashAIv1) and used it in addition to the DeepFashion dataset. They used a subset from the DeepFashion dataset including only tops clothing. Using the attributes (gender, style, color, sleeve type, and garment type), they regenerated standardized captions for DeepFashion images. To generate captions, they used a CNN encoder and RNN Decoder and jointly trained the model on both datasets. They demonstrated that dataset diversity increases caption quality for African-style images of clothing, implying that data from Western styles can be transferred. They trained their model in different ways using both Western and African datasets. The highest scores achieved were when they trained their model using both Western and African datasets, where their model achieved a BLEU-score of 65 when testing it on both datasets and 66 which is better when testing it only on the African dataset.

Another deep learning model for captioning images of clothing was developed by Dwivedi & Upadhyaya [1]. They used the merge architecture for their image captioning model, in which image features and captions are learned independently. For image features extraction, they proposed a five-layer deep Convolutional Neural Network (CNN-5), and for encoding the linguistic sequences, they used an RNN with LSTM. To train their model, they used the Fashion MNIST dataset (70,000 images). Their model achieved a BLEU-1 score of 53.48 on the test data.

Yang et al. [21] provided a large-scale dataset called FAshion CAptioning Dataset (FACAD) to study captioning images of fashion. They used a ResNet-101 as an encoder to extract the image features and LSTM as a decoder. They trained their model based on the reinforcement learning strategy to enhance the results. Furthermore, they provided two rewards to capture the semantics at the attribute level and the sentence level. The best score that their model has achieved is a BLEU-4 score of 6.8.

Cai et al. [22] created a new dataset to caption images of fashion called FACAD170K from the FACAD dataset. They also used a ResNet-101 as an encoder to extract the image features and LSTM as a decoder. They proposed a method that depends on allowing users to input preferred semantic attributes to help generate captions that fit their preferences. Their model achieved a BLEU-1 score of 46.5 and a BLEU-4 score of 19.6.

Moratelli et al. [23] presented a transformer-based captioning model that included external text memory that could be retrieved using k-nearest neighbor (KNN) searches. They encoded both the images and the text using a vision and textual transformer respectively and used the FACAD dataset to train their model. Their model achieved a BLEU-1 score of 27.3 and a BLEU-4 score of 10.6.

All these captioning models for images of clothing support only the English language, use the encoder-decoder architecture, and mostly achieve a low BLEU score compared to the size of the datasets used.

Table 2 summarizes the existing English captioning models for images of clothing.

## 3. Proposed Method

This section first provides an overview of the proposed model architecture, then presents the process of training and evaluation.

### 3.1. Model Architecture

**Figure 5:**
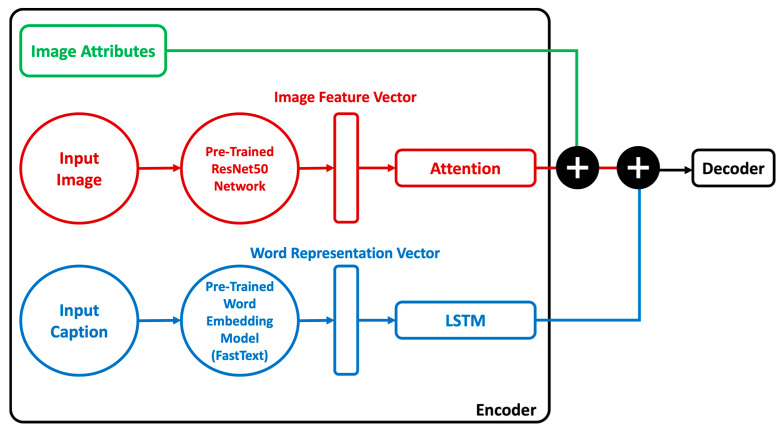
Attributes-based Arabic image captioning model architecture.

The proposed model is based on an encoder-decoder architecture. The merge architecture has been used for its simplicity and since it outperforms injection architectures. Rather than using the basic encoder-decoder architecture used by the previous researchers, in our model the attributes of the images of clothing were classified and added as inputs to the decoder to enhance Arabic caption quality. Also, an attention layer has been added after image features to allow the decoder to choose the most relevant part of the input images. The encoder of our model contains three components; an image attributes encoder, an image features encoder to extract the image features, and a linguistic sequences encoder to extract the caption features. Figure 5 shows the whole architecture of the proposed model.

Here are the details of the Attributes-Based Arabic image captioning model architecture:

#### 3.1.1. Encoder

- Image Attributes Encoder

The first step of the proposed model was classifying the attributes of the images of clothing using multi-label classification. To do that, we classified and categorized the ArabicFashionData dataset into multiple classes, each class has multiple labels that are attributes of the images of clothing it contains. In the training and validation set, we extracted the labels that represent the attributes of the image from the image path, then we converted these labels to a NumPy array and added them as input to the decoder to use it to train the decoder along with the image features and linguistic features.

- Image Features Encoder

Extracting features from an image is a key element of image captioning since it allows the system to describe a lot of the visual details in it by specifying its major patterns. Convolutional Neural Network (CNN) was used for extracting features from images. We used a pre-trained ResNet50 network as an image encoder in our model because it is trained on a very large dataset, has very high accuracy, and is available for public use [8]. Attention was applied by adding an attention layer after image features into the CNN, which aids in the concentration of selective perception. The images were fed into ResNet50, and the output was delivered to the decoder network.

- Linguistic Sequences Encoder

Linguistic representations needed to be encoded. To encode linguistic sequences of varying lengths, we used a language model based on Long Short-Term Memory (LSTM). A single-layer word embedding system was used in the LSTM model to learn the word representation. Along with the image attributes, the output from the image features encoder and linguistic sequences encoder are added as inputs to the decoder network and a final prediction is made.

#### 3.1.2. Decoder

The decoder’s task is to transform the data received from the encoder into a text sequence (generate caption). Our decoder contains a dense 128 layer with the activation function Rectified Linear Units (ReLU). The image attributes were combined with the output of the image features encoder by concatenation, and this combination was combined with the output of the linguistic sequences’ encoder by concatenation too and used as inputs to the dense layer. A softmax prediction for each word in the vocabulary is generated to be the next word in the sequence by the dense layer, and then the word with the highest probability is selected. This process is repeated until an END token is generated.

### 3.2. Model Training

We extracted the labels that represent the attributes of an image from the image path in the training and validation set. To use these attributes as input to the decoder, we converted these labels to a NumPy array. For extracting features from the images, we used a pre-trained ResNet50 network. After that, a 50% dropout was performed to avoid overfitting, and a dense 256 layer with activation function ReLU was added to transform it into a 256-element approximation of the image. Then the output was fed into reshape layer to reshape the images to enter the attention layer which expects an input sequence of a predetermined length and flattening was applied to the image features. A 50% dropout and a dense layer with activation function ReLU were added to finally produce a 128-element representation of the image. The ResNet50 network’s final output layer which contains the image classification output was removed and only the encoded image features produced by the hidden layers were used. These features were sent into the decoder as the interpretation of the image provided in the dataset. We used a pre-trained Arabic word embedding model (called FastText) to map each word/index to a 300-long vector [24]. After that, we generated an embedding matrix for each of the 60 unique words in our vocabulary, which was then fed into the model before training. We froze the pre-trained embedding layer (trainable = False) before training the model to prevent it from being changed during backpropagation. The output of the embedding layer is a 2D vector with one embedding for each word in the input sequence of words. The output was then transmitted to the LSTM layer with 256 memory units after performing a 50% dropout. Finally, a dense 128 layer was added and ReLU was the activation function used during hidden layers to overcome overfitting. Finally, using concatenation, we combined the 128-output of all three components (image attributes, image features, and linguistic features) into an output Softmax layer with the activation function ReLU that makes the final prediction for the next word in the caption over the entire output vocabulary.

### 3.3. Model Evaluation

**Figure 6:**
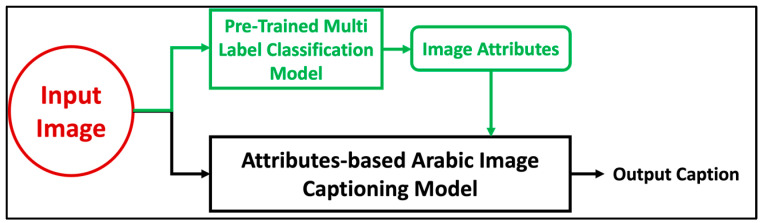
Model testing on unseen data.

To evaluate and test the model on unseen data, our trained model needs to take the image and its attributes as input and generate the caption as output as shown in Figure 6.

Because manually entering the attributes would make it impossible to accurately evaluate the model, we have built a multi-label classification model using the multi-label classified data to train and test the model.

- Multi-Label Image Classification Model (Image Attributes Classification)

The multi-label classification model was done by adopting deep residual neural networks with 50 layers (ResNet50) under the transfer learning approach to accomplish the detection task of many clothing attributes. We extracted the labels that represent the attributes of the image from the image path, then we trained the classifier on these labels with the corresponding images. This model takes the image as an input and outputs all the attributes of this image.

We used ResNet50 as the base model in our method, which had been pre-trained on the ImageNet dataset for object detection. We transferred the first 49 layers of ResNet50, which were left frozen on the multi-label classification model, using transfer learning techniques. We removed the 1000 fully connected softmax from ResNet50 and initialized a new one. Our classifier contains 64 output nodes instead of 1000 because we have 64 unique attributes. We trained a 64 fully connected sigmoid using the labeled images of clothing as input and then replaced the 1000 fully connected softmax from ResNet50 with our trained 64 fully connected sigmoid. The training and validation set of these attributes that were used to train and validate the multi-label classification model is the same that we used as input to the decoder to train our image captioning model. Adam optimizer with a learning rate of 0.0001 was used over binary cross-entropy loss function. The whole experiment was run for 2 epochs with a training batch size of 200 and a validation batch size of 200.

## 4. Experimental Setup

### 4.1. Dataset

- ArabicFashionData Dataset (AFD)

**Figure 7:**
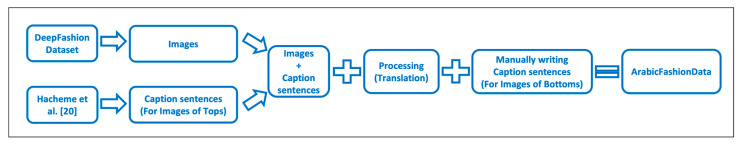
The process of creating our ArabicFashionData dataset.

Through our research reviews, we were unable to find previous use of deep learning-based Arabic image captioning in the field of clothing nor did we find an Arabic dataset of captioned images of clothing. Hence, we created an Arabic dataset for images of clothing which we named “ArabicFashionData” to evaluate our model. Figure 7 shows an overview of the process of creating the ArabicFashionData dataset.

**Figure 8:**
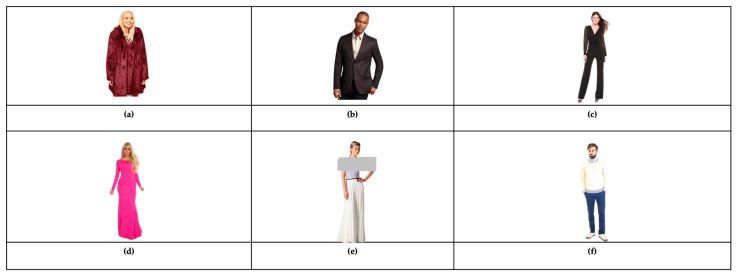
Some images with standardized caption sentences from the dataset. (**a**) The woman is wearing a red long sleeve coat. (**b**) The man is wearing a black long sleeve blazer. (**c**) The woman is wearing a long black jumpsuit with a long sleeve. (**d**) The woman is wearing a long pink dress with a long sleeve. (**e**) The woman is wearing a long white skirt. (**f**) The man is wearing a long blue pants.

The AFD dataset consists of images and a single caption for each image. The images of the AFD dataset were obtained from the DeepFashion dataset (described earlier) without their attributes, captions, or labels. The caption sentences were written in the Arabic language based on the attributes that we chose to use which are gender, garment type, color, sleeve styles, and garment length. From the DeepFashion dataset, we chose the Category and Attribute Prediction Benchmark, and we selected a subset from it. The AFD dataset included images of different types of tops such as hoodies and blazers, and different types of bottoms such as pants and skirts, in addition to dresses and jumpsuits. For the caption sentences, we translated and processed the captions produced by Hacheme et al. [20] for the images on the DeepFashion dataset to suit our work. That is because their caption sentences include the same attributes we used. So instead of writing caption sentences for each image, we ordered and translated their caption sentences to Arabic using the templates below. Since the attribute <garment length> was not included in their caption sentences, we added it manually to the garment types (dresses and jumpsuits) as shown in Figure 8c,d. For the caption sentences of images of bottoms, we wrote the caption sentences for each image of bottoms manually as shown in Figure 8e,f.

To predict captions with vocabulary that people are familiar with, the process of translating some garment types to Arabic was done based on six online shopping websites that are popular in Saudi Arabia. They are ZARA, H&M, OUNASS, 6thStreet, SHEIN, and MANGO. The garment types that were translated based on these online shopping websites were sweater, hoodie, jacket, blouse, blazer, cardigan, jumpsuit, t-shirt, dress, coat, leggings, short pants, joggers, pants, and skirt. Table 3 shows the Arabic translation of each garment type used in the dataset.

The dataset included 15 different garment types shown in Table 3, 17 different color attributes, 2 gender types, 2 garment lengths, and 3 sleeve styles.

Based on the attributes we chose to use, we wrote the caption sentences using the templates presented below in Table 4.

After choosing the attributes, we made a multi-label classification by reordering the dataset. Where each group of images that share the same attributes was classified and placed in the same class named with these attributes to use them to train the image captioning model along with the image features and linguistic features.

### 4.2. Evaluation Metric

#### 4.2.1. Multi-Label Classification Model Evaluation

The accuracy evaluation metric was used to evaluate the multi-label classification model. Accuracy is the ratio of the number of correct predictions to the total number of predictions [25]. The accuracy equation is as below:

(1) $$Accuracy=\frac{Number\;of\;correct\;predections}{Total\;number\;of\;predections\;made}$$

Our multi-label classification model has achieved an accuracy of 99% on the training data and an accuracy of 97.5% on the test data.

#### 4.2.2. Arabic Image Caption Generator Model Evaluation

In our model, captions are generated in the form of sentences. Language, on the other hand, is a subjective topic, and sentence formation based on description varies from person to person. As a result, calculating the model’s accuracy correctly becomes challenging. We used the BLEU score (Bilingual Evaluation Understudy Score) to evaluate our model. The Bilingual Evaluation Understudy (BLEU) score is the standard evaluation metric that is used to evaluate image captioning models. BLEU measures the number of words in the machine-generated caption that existed in the human reference caption. BLEU’s output is always a number between 0 and 1. The higher the value, the better the prediction. Researchers sometimes express their results in integers by converting the resulting decimal number to an integer number by multiplying the decimal number by 100. In this case, the output is a number between 0 and 100. The higher the value, the better the prediction. The formula for the BLEU score can be given as:

(2) $$BLEU=BP.\exp\left(\sum_{n=1}^{N}w_{n\;}\log p_{n}\;\right)$$

Different values of N can be used to calculate the BLEU score. N is the number of n-grams which is an expression for a group of ‘n’ consecutive words in a sentence. The default value of N is 4: BLEU-1, BLEU-2, BLEU-3, and BLEU-4 [26].

Two methods are used to transform the generated caption to a probability score for each word in the vocabulary: the greedy method and the beam search method. In the greedy method, for each time step, the word with the highest probability is chosen. In the beam search technique, which is more efficient, from a sequence of candidates, the sequence with the highest overall score is chosen [27]. The BLEU score can be calculated for both methods.

### 4.3. Experimental Settings

(1) Data Preprocessing

The dataset is processed before training the model in several steps including:

- The AFD dataset was divided into two classes, men and women. Then we randomly divided the dataset into a training set, validation set, and testing set. The training set contained 80% of all data and the rest were used for the validation set, and the testing set, each 10% of the data. Table 5 shows the summary of the ArabicFashionData dataset.

The dataset contains 79,115 images of clothing, each with a single caption sentence. The number of words in the predicted caption was limited to 10 words which is the longest caption sentence in the dataset. Additionally, the symbols <START> and <END> were added to the beginning and end of each sentence, respectively.

- The attributes of the images were preprocessed in two steps. First, we extracted the labels that represent the attributes of the image from the image path, then we converted these labels to a NumPy array.
- The images of clothing were preprocessed in two steps. We first resized the input images to 100 × 100 pixels. Then we used data augmentation on the training set of men’s clothing images to avoid the problem of unbalanced data. This was caused by the fact that men’s images and garment types were less than women’s images and garment types such as dresses, skirts, and jumpsuits and we wanted to cover all garment types. Data augmentation was done by using random transformation on the available data samples. Random horizontal, vertical flipping, rotation, shifting, and brightness were applied to the images to augment them. After augmentation, the number of images in the training set became 117,484 where 54,192 new images were added.
- A caption was preprocessed in four steps. We first tokenized the text. Tokenization divides the raw text into words separated by punctuation, special characters, or white spaces where the separators were discarded. Second, we counted the tokens where the learning data’s vocabulary became 64 words after tokenization. Third, we created index-to-word and word-to-index structures and used them to translate token sequences into word identifier sequences. Fourth, padding was done, which means padding the identifier sequences at the end with null tokens to have the inputs of the same size.

(2) Hyperparameters During Training

The model was trained for 20 epochs with an Adam optimizer with a learning rate of 0.0001 over the categorical cross-entropy loss function. The training batch size was 100 and the validation batch size was 10.

## 5. Results and Discussion

We used the greedy method and beam search technique to generate the caption. Then we calculated the BLEU score for each of them and analyzed the results to see which is better. We used the beam search technique with a beam size of 3, which means that it considers the top 3 candidate words at the first decode step. It generates three-second words for each of the first words and chooses the top three combinations of first and second words based on the additive score. After three sequences have been completed, the sequence with the highest overall score is chosen.

Given the amount of training data, the model was fit for 20 epochs. The loss computed was 0.00000009 on the training dataset and a loss of 0.000003 was on the validation dataset. Our model gave a BLEU-1 score of 88.51 in the greedy method, and a BLEU-1 score of 88.52 in the beam search technique. The results are represented in Table 6.

We also implemented the common model which uses only image features and linguistic features to train the model and evaluated it using our dataset. Then we compared the results. Table 7 compares our attributes-based model with the common model.

Based on the previous results, we found that training the model using image attributes along with image features and linguistic features achieved higher results than using only image features and linguistic features to train the model.

Table 8 compares our attributes-based model with other state-of-the-art models. In particular, the comparison includes (i) generic models trained with an Arabic dataset that are not related to fashion, and (ii) fashion models trained with an English dataset, since the use of deep learning-based Arabic image captioning in the field of fashion has not been found. For the first category, the comparison includes [14,16] which studied generating root words rather than actual sentences using compositional architecture. It also includes [3,15,17] which used an encoder-decoder architecture for generating Arabic captions using either an inject model or a merge model. For the second category, it considers the models developed for the fashion domain which includes [20] that explored training the model using diversity datasets [1] which proposed a smaller 5-layer Convolutional Neural Network (CNN-5) to extract image features, Semantic Rewards guided Fashion Captioning (SRFC) [21]. It also includes [22] which explored taking the semantic attributes from the users, and [23] which developed a transformer-based model.

As can be observed, according to BLEU, the proposed approach performs better than all of the methods that were compared. This demonstrates the success of training the model utilizing image attributes in addition to the image features and linguistic features and represents an improvement of more than 30 BLEU points over the model that does not use attributes and other related models. For example, our model outperforms [22] with more than 40 BLEU points and [23] with more than 60 BLEU points.

## 6. Conclusions

Image captioning is a difficult computer vision challenge. Future studies will benefit from this study because there is still a lot of work to be done in order to produce Arabic captions for digital images. The major goal is to have these automatically generated captions precise enough to be considered human-like. In this work, we created an Arabic dataset for captioning and classifying images of clothing and made it publicly available.

Furthermore, we proposed an approach that depends on passing the image attributes as input along with image features and linguistic features to train the model. For validation, a model for multi-label classification was developed to output attributes that were used as input to the trained model to generate the final caption for the input image. In terms of the BLEU score, the proposed model performs better than state-of-the-art models. Nonetheless, there is still plenty of potential for further improvement.

In conclusion, our work highlights the advantages of using classification when applying image captioning to a specialized field, like the fashion one. As a part of future work, extra attributes will be added to accurately describe the images of clothing, and textile attributes such as fabric type will be taken into consideration. Moreover, other methods will be taken into consideration such as Landmark Detection [28], and other kinds of neural networks such as Transformers [29].

## Figures and Tables

**Table 1:** Summary of Arabic image captioning approaches.

Reference	Architecture	Image Model	Language Model	Attention	Dataset	Evaluation Metric (BLEU-1)
[14]	Compositional	R-CNN	RNN-Deep Belief Network (DBN)	No	ImageNet (10,000) Aljazeera News (100,000)	34.8
[15]	Encoder- Decoder	CNN	RNN-LSTM	No	Subset of Flickr8k (2000)	34.4
[3]	Encoder- Decoder	CNN	RNN-LSTM	No	MSCOCO dataset (1166) Flickr8k datasets (2261)	46
[16]	Compositional	R-CNN	RNN-LSTM	No	Flickr8k (8092)	65.8
					Middle Eastern newspapers (405,000)	55.6
[17]	Encoder-Decoder	CNN	RNN-LSTM	No	Flickr8k (8092)	33

**Table 2:** Summary of captioning models for images of clothing.

Reference	Architecture	Image Model	Language Model	Attention	Dataset	Evaluation Metric (BLEU-1)
[19]	Encoder- Decoder	CNN	RNN-LSTM	No	DeepFashion (20,096)	-
[20]	Encoder- Decoder	CNN	RNN-LSTM	No	DeepFashion+ InFashAIv1	66
[1]	Encoder- Decoder	CNN	RNN-LSTM	No	Fashion MNIST dataset (70,000)	53.48
[21]	Encoder- Decoder	CNN	RNN-LSTM	Yes	FACAD dataset (993,000)	-
[22]	Encoder- Decoder	CNN	RNN-LSTM	Yes	FACAD170K dataset (178,862)	46.5
[23]	Encoder- Decoder	Transformer Neural Network (TNN)	Transformer Neural Network (TNN)	Yes	FACAD dataset (993,000)	27.3

**Table 3:** The Arabic translation of all used garment types.

Garment Types	Arabic Translation
Sweater	سترة
Hoodie	هودي
Jacket	جاكيت
Blouse	بلوزة
Blazer	بليزر
Cardigan	كارديجان
Jumpsuit	جمبسوت
T-Shirt	قميص
Dress	فستان
Coat	معطف
Leggings	بنطلون ليقنز
Short pants	بنطلون شورت
Jogger	بنطلون رياضي
Pants	بنطلون
Skirt	تنورة

**Table 4:** The templates used to write the caption sentences.

Template	Arabic	English	Example
Top clothing	<garment type> ترتدي/يرتدي <gender> الأكمام <sleeve style> اللون و <color>	The <gender> is wearing a/an <color><sleeve style><garment type>	Figure 8a,b
Dresses and jumpsuits	<garment length><garment type>ترتدي<gender> الأكمام <sleeve style> اللون و <color>	The <gender> is wearing a <garment length><color><garment type> with a <sleeve style>	Figure 8c,d
Bottom clothing	<garment type> ترتدي/يرتدي <gender> اللون <color><garment length>	The <gender> is wearing a <garment length><color><garment type>	Figure 8e,f

**Table 5:** ArabicFashionData dataset split.

	**Dataset Split**			
	Training (80%)	Validation (10%)	Testing (10%)	Total
Images	63,292	7911	7912	79,115

**Table 6:** BLEU score for the proposed model.

Search Type	BLEU-1	BLEU-2	BLEU-3	BLEU-4
Greedy	88.51	83.87	81.14	74.48
Beam	88.52	83.87	81.15	74.50

**Table 7:** Comparing the proposed model with the common model using the BLEU score.

Model	Search Type	Dataset	Language	BLEU-1	BLEU-2	BLEU-3	BLEU-4
Common model (without attributes)	Greedy	ArabicFashionData	Arabic	48.20	36.14	30.04	13.39
	Beam			56.53	47.10	43.42	30.98
Our attributes-based model	Greedy	ArabicFashionData	Arabic	88.51	83.87	81.14	74.48
	Beam			88.52	83.87	81.15	74.50

**Table 8:** Comparing our model with different models using the BLEU score.

Search Type	Model	Dataset	Language	BLEU-1	BLEU-2	BLEU-3	BLEU-4
-	[14]	ImageNet + Aljazeera News	Arabic	34.8	-	-	-
-	[16]	Flickr8k	Arabic	65.8	-	-	-
-	[15]	Subset of Flickr8k	Arabic	34.4	-	-	-
-	[17]	Flickr8k	Arabic	33	-	-	-
-	[3]	MSCOCO + Flickr8k	Arabic	46	-	-	-
-	[20]	DeepFashion + InFashAIv1	English	66	-	-	-
-	[1]	Fashion MNIST dataset	English	53.48	23.57	14.88	6.6
-	SRFC [21]	FACAD	English	-	-	-	6.8
Beam	[22]	FACAD170K	English	46.5	-	-	19.6
Beam	[23]	FACAD	English	27.3	-	-	10.6
Greedy	Our attributes-based model	ArabicFashionData	Arabic	88.51	83.87	81.14	74.48
Beam	Our attributes-based model	ArabicFashionData	Arabic	88.52	83.87	81.15	74.50

## Data Availability

Data supporting reported results can be found here: https://github.com/Rasha-AlMalki/ArabicFashionData_Dataset.git (accessed on 2 March 2023). The dataset that was used to create the dataset used in this study is a public dataset DeepFashion [18].
